# VanA-*Enterococcus faecalis* in Poland: hospital population clonal structure and *vanA* mobilome

**DOI:** 10.1007/s10096-022-04479-4

**Published:** 2022-09-03

**Authors:** Ewa Wardal, Dorota Żabicka, Waleria Hryniewicz, Ewa Sadowy

**Affiliations:** 1 Department of Molecular Microbiology, National Medicines Institute, Chełmska 30/34, 00-725 Warsaw, Poland; 2 Department of Epidemiology and Clinical Microbiology, National Medicines Institute, Chełmska 30/34, 00-725 Warsaw, Poland

**Keywords:** *Enterococcus faecalis*, VRE, Plasmid, HAI, Tn*1546*

## Abstract

The aim of our study was to characterize the epidemiological situation concerning nosocomial vancomycin-resistant *Enterococcus faecalis* of VanA-phenotype (VR*Efs*-VanA) in Poland by investigating their clonal relationships and the *vanA*-associated mobilome. One-hundred twenty-five clinical isolates of VR*Efs*-VanA collected between 2004 and 2016 were studied by phenotypic assays, multilocus sequence typing (MLST), pulsed-field gel electrophoresis (PFGE), PCR detection of plasmid-specific genes, and Tn*1546* structure and localization mapping. Selected isolates were subjected to PFGE-S1, Southern hybridization, genomic sequencing and conjugation experiments. The majority of isolates (97.6%) belonged to clonal complexes CC2 and CC87 of *E. faecalis*. All isolates were resistant to vancomycin and teicoplanin, and resistance to ciprofloxacin and aminoglycosides (high level) was very prevalent in this group. VanA phenotype was associated with 16 types of Tn*1546*, carrying insertion sequences IS*1216*, IS*Efa4*, IS*1251* and IS*1542,* located on *repUS1*_pVEF1_, *rep1*_pIP501_, *rep2*_pRE25_, *rep9*_pAD1/pTEF2/pCF10_ and *rep6*_pS86_ replicons. The most common Tn*1546* B- and BB-type transposons, harbouring one or two copies of IS*1216*, were inserted between *rep18a*_p200B_ and *repUS1*_pVEF1_ genes and located on ~ 20 kb and 150–200 kb plasmids. VR*Efs-*VanA in Poland represent a polyclonal group, indicating a number of acquisitions of the *vanA* determinant. The *repUS1*_pVEF1_-*vanA* plasmids, unique for Poland, were the main factor beyond the acquisition of vancomycin resistance by *E. faecalis,* circulating in Polish hospitals.

**Supplementary Information:**

The online version contains supplementary material available at 10.1007/s10096-022-04479-4.

## Introduction

*Enterococcus faecalis* is the most common species of enterococci, widely distributed in humans, animals and the environment. It also significantly contributes to the overall number of healthcare-associated infections (HAIs) caused by antimicrobial resistant bacteria [1]. Population analyses of hospital *E. faecalis* based on multilocus sequence typing (MLST) revealed the existence of a few clonal complexes (CCs) dominating among clinical isolates [2–5]. Three of these complexes, CC2, CC9 and CC87, include invasive, multi-drug resistant isolates frequently obtained from hospitalized patients but found also in faecal carriage [6].

The majority of antibiotics, including aminoglycosides, cephalosporins, fluoroquinolones, tetracyclines, phenicols, macrolides, lincosamides and streptogramins, are often excluded from therapy of *E. faecalis* infections due to various intrinsic and acquired resistance mechanisms [7]. Thus, glycopeptides (vancomycin and teicoplanin) represent important drugs in the treatment of enterococcal infections. Resistance to these compounds in *E. faecalis* is being reported in Europe, although with much lower frequency (1.1%) than in *Enterococcus faecium* (19.0%) [8]. Recently, in Poland, 2.5% of all *E. faecalis* invasive infections are caused by vancomycin-resistant *E. faecalis* (VR*Efs*) [1]. The appearance of VR*Efs* poses a therapeutic challenge and a risk for transmission of glycopeptide-resistance to other hospital pathogens, in particular to *Staphylococcus aureus* [9, 10].

The *vanA* gene cluster, which confers resistance to both vancomycin and teicoplanin (VanA phenotype), is predominantly associated with different variants of Tn*1546-*type transposons, which result from point mutations, deletions and activity of insertion sequences [11–13]. In Poland, the first VR*Efs* isolate with the *vanA* determinant was observed already in 1998 [14]. Two plasmid families associated with VanA determinants have been described for *E. faecalis,* including broad host Inc*18* plasmids and pheromone-responsive plasmids, the latter restricted almost exclusively to this species [10, 15, 16]. Multiple acquisitions and occasional losses of *vanA*-carrying mobile genetic elements (MGEs), variation of Tn*1546* insertion sites and clonal expansion of particular strains often following *vanA* acquisition describe the dynamics of hospital VR*Efs* [17]. However, the knowledge about worldwide diversity and activity of VanA-MGEs in *E. faecalis* and the global population structure of VR*Efs* is still far from being complete.

After the first report of VR*Efs-*VanA in hospital settings in Poland in 1998 [14], these pathogens re-appeared in 2004 and since then are regularly submitted to the National Reference Centre for Susceptibility Testing (NRCST). The aim of this study was to provide the in-depth phenotypic and molecular characteristics of VR*Efs*-VanA clinical isolates from 2004 to 2016 collected by the NRCST, with the special focus on its population structure and *vanA*-associated MGEs in order to better understand the epidemiology of these pathogens in our country.

## Materials and methods

### Bacterial isolates and phenotypic testing

The study comprised 125 consecutive, non-repetitive (1 isolate per patient) VR*Efs*-VanA isolates received by the NRCST from 38 hospitals in 25 cities in Poland over the period 2004–2016, including fifteen isolates from year 2004 [3, 18] and the 1207/14 isolate from 2014 [19]. Twelve isolates (9.6%) were from invasive infections (all isolates from blood), 61 isolates (48.8%) were from non-invasive infections (23, 15 and 23 isolates from urine, wounds and other sources, respectively) and 52 isolates (41.6%) represented faecal carriage. Additionally, the first Polish ST87 VR*Efs*-VanA isolate from 1998 [14] was used for comparative purposes. Presumable outbreaks were defined as appearance of two or more VR*Efs*-VanA infections in a single hospital at the same or narrow time [20]. Antimicrobial susceptibility was tested using gradient tests for daptomycin, teicoplanin and vancomycin (BioMérieux, Marcy-l’Etoile, France), and by a broth microdilution method for the remaining compounds (ISO 20776–1 standard). The results were interpreted following the European Committee on Antimicrobial Susceptibility Testing (EUCAST)-approved breakpoints for ampicillin, ciprofloxacin, gentamicin, streptomycin, vancomycin, teicoplanin, tigecycline and linezolid [21], and the Epidemiological Cut-Off (ECOFF) values for compounds without defined breakpoints, such as penicillin, tetracycline, daptomycin and chloramphenicol (http://mic.eucast.org/Eucast2/, last accessed 10^th^ March 2022). The isolates were examined for the production of cytolysin using a hemolytic test [22]. In brief, bacterial cultures were streaked on THB agar with 5% of horse blood, incubated 24–72 h in 5% CO2 at 37 °C and examined for the presence of clearing zones around the colonies. The production of aggregation substance (AS) was assessed by observation of cell clumping in the presence of sex pheromone from culture supernatants of the OG1X strain of *E. faecalis* [23].

### DNA isolation and PCR detection of van determinants

Total DNA of isolates was extracted using the Genomic DNA Prep Plus kit (A&A Biotechnology, Gdansk, Poland). The detection of the *vanA* and *vanB* genes was performed by PCR [24, 25] with *E. faecium* BM4147 and *E. faecalis* V583 as positive controls, respectively.

### Genotyping of isolates by MLST and PFGE

MLST was performed as described [26] using the web-accessible database https://pubmlst.org/organisms/enterococcus-faecalis (last accessed 18^th^ October 2021) to establish alleles and sequence types (STs) [27]. STs were grouped into CCs by the comparative eBURST analysis performed against the whole *E. faecalis* MLST database (https://pubmlst.org; last accessed 31^st^ March 2020). The MLST data for three isolates (6210/09, 6432/09 and 6878/09) were reported previously [28] and used in the current analyses. Pulsed-field gel electrophoresis (PFGE) was performed according to de Lancastre et al. [29] for agarose plugs preparation, followed by the procedure of Clark et al. [30] for total genomic DNA purification. Purified DNA in plugs was digested with the *Sma*I restriction enzyme (Fermentas, Vilnius, Lithuania). Electrophoresis was performed at 14 °C for 22 h with pulse time 1–30 s at 6 V/cm^2^ in 0.5 × TBE buffer. Lambda PFG Ladder (New England Biolabs, Ipswich, MA) was used as a DNA size standard. The Dice similarity coefficient (1% optimization, 1% tolerance, 1% tolerance change) and the unweighted pair-group mean arithmetic method (UPGMA) in the Bionumeric software (Applied Maths, Kortrijk, Belgium) were used to analyse PFGE-banding patterns, with the 85% similarity cut-off value defining a PFGE type (PT).

### Tn1546 typing and localization

**Fig. 1:**
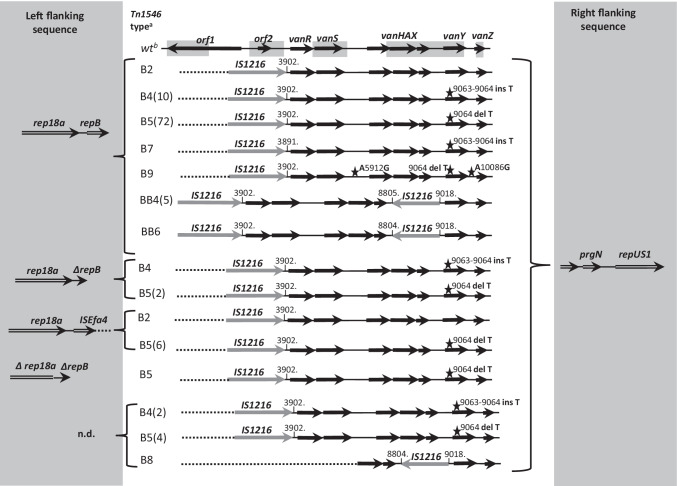

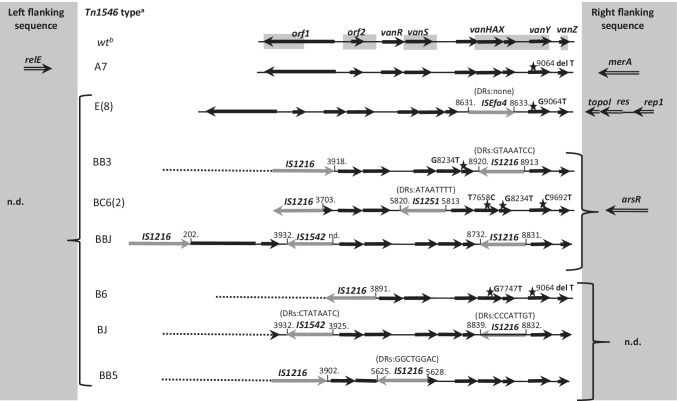
Diversity of Tn*1546* transposon types among *E. faecalis* VanA isolates. Black arrows, transposon genes; stars, positions of point mutations; areas of the transposon analysed by PCR mapping and sequencing shadowed; the A5912G and A10086G point mutations detected on Illumina reads; dashed lines, deletions in the left arm of the transposon; grey arrows, ISs; DR, direct repeats; ins, insertion; del, deletion; n.d.,– not determined; ^a^ number of isolates, if larger than one, are given in brackets; ^b^ reference Tn*1546* (M97297). Nucleotide positions correspond to the reference Tn*1546* transposon

Tn*1546* transposon structure was investigated by PCR mapping and sequencing (Supplementary Table 1 and references therein) of selected regions encompassing 7571 bp out of 10,851 bp, i.e. ~ 70% of the transposon (Fig. 1.). The Tn*1546* sequence of *E. faecium* BM4147 (GenBank acc. no.: M97297) [11] was used as a wild-type reference (A1 type). The nomenclature of Tn*1546*-type transposons in the current study was based on the alphanumeric code used previously for Polish *E. faecium* VanA [31], according to which the ‘A’ types referred to transposon variants affected only by point mutations; the ‘B’ types contained 1–2 copies of IS*1216* (B and BB types); the C, E and J types carried IS*1251*, IS*Efa4* and IS*1542* elements, respectively. Transposons with more than one IS type were described by a two- or three-letter code (e.g. ‘BC’ for transposon with both IS*1216* and IS*1251*). The Arabic numerals indicated the presence of point mutations compared to the wild-type A1 transposon and/or differences in orientation of ISs and the localization of their insertion sites (e.g. B2-B9). Primers used for PCR targeting junctions between Tn*1546* and its insertion sites were designed based on 1207/14 isolate [19] and genomic sequences obtained in this study (Supplementary Table 1).

### Plasmid analysis

Detection of 19 *rep* families and the unique *rep*_pMG1_ gene was performed according to Jensen et al. [32]. PCR primers for amplification of *repUS1*_pVEF1_ and *rep18a*_p200B_ not included in the original scheme were designed based on the 1207/14 genome [19]. PCR detection of *asa*, *cylA*, *bee*, *rep*_pLG1,_ *repUS1*_pVEF1_, *rep18a*_p200B_, plasmid addiction systems and relaxase genes was performed as described (Supplementary Table 1 and references therein). A few randomly selected PCR products were sequenced to confirm the results for each of the detected genes. For plasmid profiling and hybridization analyses, DNA in agarose plugs was obtained as described above [28, 29], treated with S1 nuclease (Takara Bio, Japan) and separated by PFGE [33] followed by blotting onto the Hybond membrane (GE Healthcare, Buckinghamshire, UK) by a capillary transfer. The size of plasmid bands was estimated according to Lambda PFG Ladder and plasmids < 50 kb were additionally compared to the 21.6 kb p1207_4 plasmid of the 1207/14 isolate [19]. Hybridization was carried out using the Amersham ECL Random-Prime Labelling and Detection System (GE Healthcare) with the *vanA, rep1*_pIP501_, *rep2*_pRE25_, *rep6*_pS86_, *rep8*_pAM373_, *rep9*_pAD1/pTEF2/pCF10_, *rep13*_pC194_, *rep18b*_pEF418_, *repUS1*_pVEF1_, *par*, *rel*_pAD1_, *rel*_pCIZ2_, *rel*_pAMalpha_, *asa* and *cylA* probes.

### Conjugation

Mating experiments were performed with *E. faecalis* OG1RF and *E. faecium* 64/3 recipients according to the procedure developed for strains with a low transfer efficiency [34]. To this end, overnight liquid cultures of donors and recipients were mixed in a 1:1 ratio, spread on BHI agar plates and incubated at 37 °C for 24 h. Bacterial cells were then transferred onto a selective medium (BHI agar with 32 mg/l vancomycin, 64 mg/l fusidic acid and 64 mg/l rifampicin) and incubated at 37 °C for 48 h.

### Whole-genome sequencing and data analysis

For whole genome sequencing (WGS), total DNA of selected isolates was obtained using the Genomic Mini AX Bacteria Kit (A&A Biotechnology, Gdynia, Poland) according to the manufacturer’s instructions and sequencing was carried out on the Illumina MiSeq Platform with the PE300 mode (Illumina Inc., San Diego, CA) as an external service (Genomed S.A., Warsaw, Poland). Reads were trimmed with Cutadapt v 1.16 [35], assembled using Spades v 3.11.1 [36] and annotated with PROKKA 1.11 [37]. Supplementary manual BLASTx analyses (https://blast.ncbi.nlm.nih.gov/) were used when appropriate. Complete genomic sequences of *E. faecalis* were downloaded from GenBank (23^rd^ February 2022), analysed using mlst [https://github.com/tseemann/mlst; 8^th^ March 2022 date last accessed] and annotated with PROKKA. Core genome alignments were obtained with Roary [38] and used in RAxML [39] for construction of Maximum Likelihood (ML) trees. For identification of *vanA-*plasmids of *E. faecalis* in GenBank the complete Tn*1546* sequence was used as a query for blastn search of the nt/nr database (as of 29^th^ October 2021), limited by: organism: “Enterococcus faecalis (taxid:1351)” and the hits with the query coverage over 50% were included in the final set. The PlasmidFinder 2.0.1 [40] and ResFinder 3.0 [41] services (both last accessed 25^th^ February 2022) were used to identify known plasmid replicon families and resistance genes, respectively, in genomic data. Conjugation transfer-associated regions were detected by oriTFinder [42]. The p1207_4 plasmid sequence was visualized using the BLAST Ring Image Generator (BRIG, http://brig.sourceforge.net) [43].

### Statistical analysis

Chi-squared test was used to assess the differences of distributions, with *p* ≤ 0.05 considered significant.

### Accession numbers

The whole-genome shotgun project has been deposited at DDBJ/ENA/GenBank under the BioProject number PRJNA731638. The accession numbers of draft genome sequences described in this paper are listed in Table 2. The complete genome sequence of 1207/14 isolate of VR*Efs*-VanA published recently [19] can be found in the DDBJ/ENA/GenBank under the accession numbers CP075604 (chromosome), CP075605 (p1207_1), CP075606 (p1207_2), CP075607 (p1207_3), CP075608 (p1207_4), CP075609 (p1207_5) and CP075610 (p1207_6).

## Results

### Population structure of Polish VREfs-VanA

**Fig. 2:**
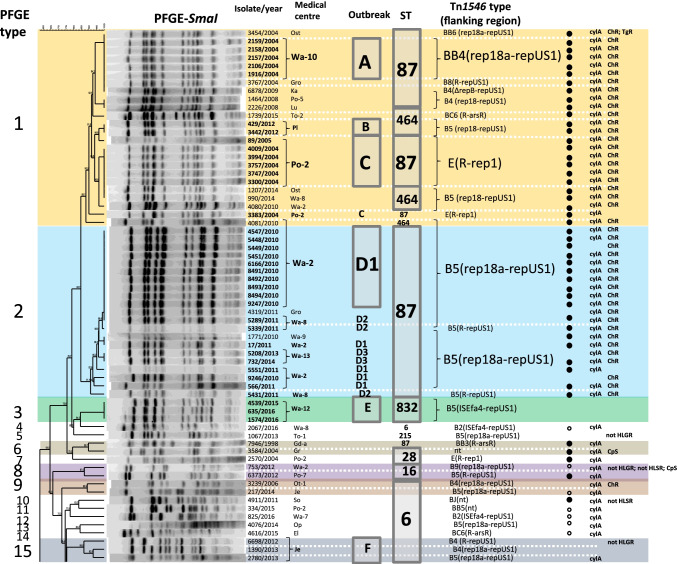

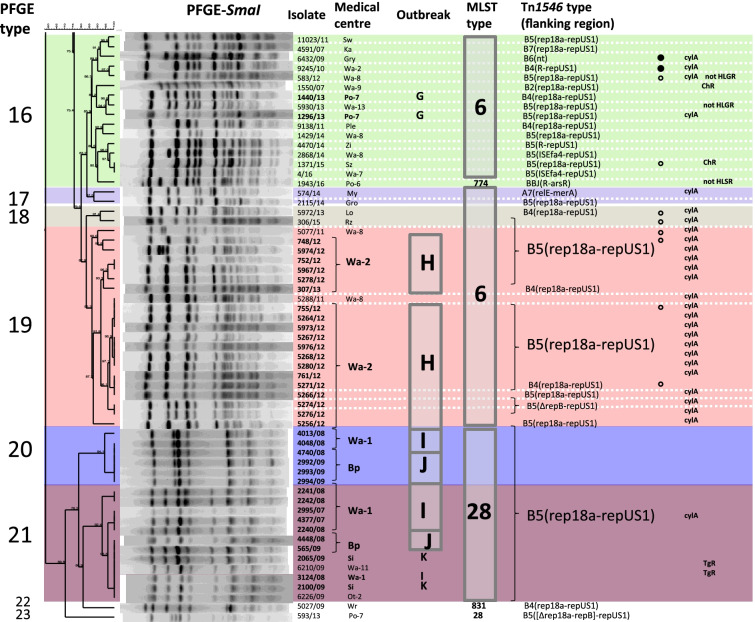
PT-based dendrogram of VR*Efs*-VanA in Poland (*n* = 125) collected during 2004–2016. *Bp*, Biała Podlaska; *El*, Elbląg; *Gd* Gdańsk,; *Gr*, Grajewo; *Gro,* Grodzisk Mazowiecki; *Gry*, Gryfice; *Je*, Jelenia Góra; *Ka,* Katowice; *Lo,* Łódź; *Lu*, Lublin; *My*, Myślenice; *Op,* Opole; *Ost,* Ostrów Mazowiecki; *Ot,* Otwock; *Pl,* Płock; *Ple*, Pleszew; *Po,* Poznań; *Rz,* Rzeszów; *Si*, Siedlce; *So*, Sosnowiec; *Sw*, Świdnica; *Sz*, Szczecin; *To*, Toruń; *Wa,* Warszawa; *Wr,* Wrocław; *Zi,* Zielona Góra; the city abbreviation is followed by the centre number; presumable outbreaks designated with letters A-K; TgR, isolate resistant to tigecycline; ChR, isolate resistant to chloramphenicol; CpS, isolate susceptible to ciprofloxacin; not HLSR, isolate susceptible to high concentration of streptomycin; not HLGR, isolate susceptible to high concentration of gentamicin; only phenotypes that differentiate the isolates are shown. Filled and empty circles indicate strong and weak haemolysis activity, respectively. The first Polish VR*EFs*-VanA isolate (7946/1998) included for comparative purposes

During the study period (2004–2016), two cities, Warsaw and Poznań, were most affected by VR*Efs*-VanA, with 86 isolates (68.8%) originating from 14 different medical centres and causing several outbreaks (outbreaks A, C, D1-D3, E, G, H and I in Fig. 2). The remaining 39 isolates were responsible for four small outbreaks in Pl, Je, Bp and Si hospitals (outbreaks B, F, J and K, respectively) as well as 26 sporadic cases in other Polish cities. MLST and PFGE discerned nine STs and 23 different PTs within the studied population, respectively (Fig. 2.). In the MLST analysis, the vast majority of isolates belonged to two clonal complexes, CC87 (*n* = 71; mainly STs 87 and 28) and CC2 (*n* = 51; solely ST6). Three isolates belonged to distinct STs 16 and 215. In the PFGE analysis, six most prevalent PTs such as 1, 2, 16, 19, 20 and 21 were characteristic for 100 isolates (80.0%) and grouped 70 out of 76 (92.1%) isolates presumably involved in outbreaks. The most prevalent PT1 (*n* = 24) was associated with ST87 (*n* = 17) and ST464 (*n* = 7) isolates, both belonging to CC87. Other numerous PTs also highly correlated with specific STs, i.e. PT2 corresponded solely to ST87, PT16 and PT19 were present among ST6 isolates while PT20 and PT21 belonged exclusively to ST28.

The first Polish VR*Efs*-VanA was isolated in a Gd medical centre in 1998 [14] and belonged to CC87 (ST87/PT6). VR*Efs*-VanA remained then absent in Poland until 2004, when the first CC87 outbreak occurred in the Wa-10 medical centre, involving five ST87/PT1 isolates (outbreak A in Fig. 2). Single ST87/PT1 isolates were obtained in the same year also from two other centres (Ost, Gro). Another outbreak of ST87/PT1 VR*Efs*-VanA (7 isolates) took place in the Po-2 medical centre also in 2004 (outbreak C). The ST87/PT2 isolates (*n* = 17) caused outbreaks in 2010–2011 in the Wa-2 and Wa-8 medical centres (outbreaks D1 and D2) and occasionally ST87/PT2 VR*Efs*-VanA appeared also in Wa-9, Wa-13 and Gro hospitals until 2014. In parallel, a multicenter spread of ST28 isolates (*n* = 18 isolates) belonging to related PT20 and PT21 caused outbreaks in the Wa-1, Bp and Si hospitals during 2007–2009 (outbreaks I, J and K).

While CC87 predominated in Polish hospitals during 2004–2010 (52 out of 56 isolates from this period), isolates belonging to CC2/ST6 constituted the majority of VR*Efs*-VanA isolates during the next six years (46 out of 68 isolates from 2011 to 2016). The first, sporadic ST6/PT19 isolate was observed in the Wa-8 medical centre in 2011 and likely this strain was transferred to Wa-2, where representatives of ST6/PT19 were responsible for the large hospital outbreak H (19 isolates) in the 2011–2012 period. CC2 isolates were also detected in other centres in Warsaw as well as in other cities during 2007–2016, in association with PT15 (*n* = 3; the outbreak F in Je), PT16 (*n* = 16) and several sporadic PTs (Fig. 2). The group of ST6/PT16 isolates was associated with sporadic isolations except for a small outbreak G (2 isolates) in the Po-7 medical centre in 2013.

### Phenotypic characteristics of the collection and main CCs

The MIC values of glycopeptides ranged from 128 to > 256 mg/l for vancomycin and from 8 to > 256 mg/l for teicoplanin in the studied population. All isolates carried *vanA* and lacked *vanB*. Resistance to ciprofloxacin, high level gentamicin and high level streptomycin resistance were very common in the whole collection (99.2%, 96.0% and 97.6%, respectively). Three isolates were resistant to tigecycline. All the isolates were susceptible to ampicillin and linezolid, and exhibited MIC values below the ECOFFs for penicillin and daptomycin. The MIC values for tetracycline and chloramphenicol were above the corresponding ECOFFs in the case of all isolates and 46 (36.8%) isolates, respectively (http://mic.eucast.org/Eucast2/, last accessed 15^th^ July 2020). Haemolysis was typical for 63 isolates (50.4%), among which 48 isolates displayed a strong haemolysis and 15 isolates displayed a weak haemolytic activity (Fig. 2). All but two haemolytic isolates carried the *cylA* gene (Fig. 2). The AS gene (*asa*) was detected in 108 isolates (86.4%); however, only eight isolates were positive in clumping test in the culture supernatant of the OG1X strain of *E. faecalis*. Two main CCs, 6 and 87, did not show significant differences concerning antimicrobial susceptibility phenotypes except chloramphenicol resistance, which was more prevalent in CC87 (59.7% of this CC, compared to 5.9% of CC2, *p* < 0.0001). The majority of isolates with strong haemolysis belonged to CC87 (*n* = 44; *p* < 0.0001), while weak haemolysis was associated with CC2 (*n* = 14; *p* = 0.0007). Haemolysis-negative isolates (*n* = 62) belonged to both clonal complexes and the presence of *cylA* was observed for 22 of these isolates, including mainly the outbreak H (Fig. 2).

### Tn1546-type transposon diversity

Tn*1546*-typing distinguished seven transposon types A, B, BB, BC, BJ, BBJ and E, which included 15 subtypes (Fig. 1). The vast majority, i.e. 114 isolates carried Tn*1546* of various B-types, disrupted by (i) one or two copies of IS*1216* (subtypes B2, B4-B9 and BB4-BB6); (ii) IS*1216* and IS*1251* (subtype BC6); and (iii) IS*1216* and IS*1542* (subtypes BJ and BBJ). Eight isolates carried E-type with IS*Efa4* insertion and a single isolate possessed transposon without ISs, differing from the wild-type Tn*1546* by the presence of a point mutation (the A7 subtype). Apart from the presence of various ISs, the variability of Tn*1546* was also associated with the presence of point mutations, insertions and deletions. The IS*1216* activity caused deletions of different size in the regions adjacent to the IS insertion site in the majority of transposon types (B2-B9, BB4-BB6, BC6, BJ, BBJ). Five different nucleotide substitutions (G7747T, T7658C, G8234T, G9063T, C9692T) described previously [31, 44–46] and two novel ones (A5912G, A10086G) were present in subtypes B6, B9, BC6 and E. Insertion/deletion of a single T nucleotide within the poly-T tract in the *vanY* gene (nt 9063–9071) occurred in the B4, B5, B6, B7 and B9 subtypes, resulting in translational frameshifts and a truncated VanY. For a single isolate, Tn*1546* type could not be defined due to problems with PCR amplification of parts downstream of the *vanA* gene.

The first VR*Efs*-VanA from 1998 harboured the BB3-type Tn*1546*, not observed in any later isolate. BB4- and E-types of Tn*1546* were typical for isolates causing the early CC87 outbreaks A and C (Fig. 2) in the Wa-10 and Po-2 medical centres, respectively. The most prevalent subtype B5 (84 isolates, 67.2%) was present in isolates belonging to both CC2 and CC87, occurring predominantly in Warsaw hospitals. B5 was associated with several outbreaks (B, D1-D3, E, H, I, J, K in Fig. 2) as well as with sporadic isolations. The second most prevalent subtype B4 (13 isolates) varied from B5 by a single T insertion within the poly-T tract in the *vanY* gene and was typical for sporadic cases in different cities during the 2008–2013 year period. The remaining Tn*1546* types (1–2 isolates/type) were sporadically detected among 14 isolates not involved in outbreaks.

### VREfs-VanA plasmidome, vanA-plasmids, their transfer and epidemiology

PCR screening for plasmid replicon types was performed according to the original typing scheme for Gram-positive bacteria [32] and additionally included detection of *repUS1*_pVEF1_, *repUS11*_pTEF3_ and *rep18a*_p200B_. The most frequent plasmid replicons were *rep9*_pAD1/pTEF2/pCF10_*,* characteristic for pheromone-responsive plasmids, *rep18a*_p200B_ of Rep_3 theta plasmids and *repUS1*_pVEF1_ from the Inc*18* group of plasmids (Table 1). The majority of *rep* genes was evenly distributed in both CC87 and CC2, with the exception of *rep13*_pC194_, exclusively associated with CC87, and *rep6*_pS86_, significantly overrepresented in this CC (38.0% of CC87 compared to 5.9% of CC2, *p* = 0.0001). The *rep17*_pRUM_ gene was detected only in the first Polish VR*Efs*-VanA isolate from 1998. Analysis of the distribution of plasmid mobilization and addiction systems showed the abundance of the MOB_C2_ relaxase gene (122 isolates) and the *par* addiction system (56 isolates), characteristic for the pAD1 pheromone-responsive plasmid [47, 48]. Other detected relaxase genes included two MOB_P7_ genes of pCF10 and pCIZ2 [48, 49], and the MOB_V_ gene, typical for pAMalpha [48, 50] (9, 106 and 47 isolates, respectively).

**Table 1:** Distribution of *rep* genes among Polish VanA-*E. faecalis*

Rep superfamily (number of isolates)	*rep* family	Number of isolates	CCs/distinct STs (number of isolates)	Year period
RepA_N (122)	*rep8*_pAM373_	3	CC87(2), CC2(1)	2004–2013
	*rep9*_pAD1/pTEF2/pCF10_	122	CC87(72), CC2(48), ST16(1), ST215(1)	1998–2016
	*rep17*_pRUM_	1	CC87(1)	1998
Inc18 (31)	*repUS1*_*pVEF1*_	109	CC87(61), CC2(45), ST6(2), ST215(1)	2004–2016
	*rep1*_pIP501_	10	CC87(8), CC2(2)	2004–2011
	*rep2*_pRE25_	24	CC2 (13), CC87 (10), ST215(1)	2004–2016
	*rep*_pMG1_	2	CC2(2)	2012,2016
Rep_3 (46)	*rep6*_pS86_	30	CC87(27), CC2(3)	2004–2016
	*rep18a*_*p200B*_	112	CC87(60), CC2(49), ST16(2), ST215(1)	2004–2016
	*rep18b*_pEF418_	20	CC87(10), CC2(10)	2005–2016
RCR (67)	*rep7*_pT181_	65	CC87(49), CC2(16)	2004–2015
	*rep13*_pC194_	19	CC87(19)	1998–2009

**Fig. 3:**
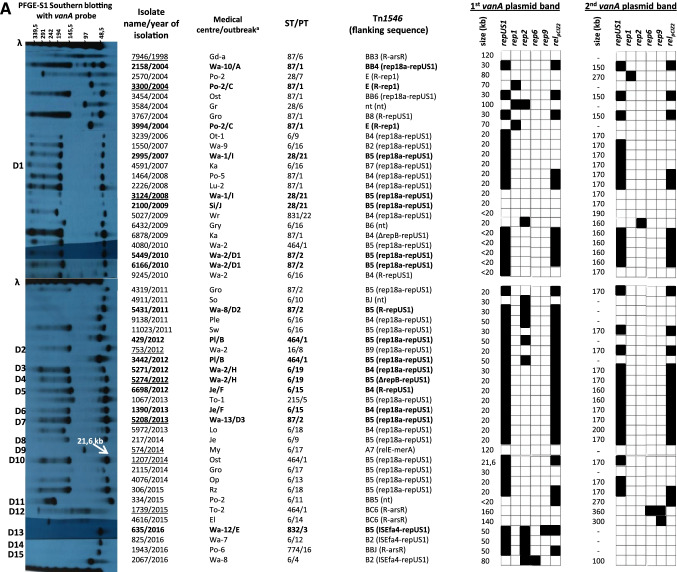

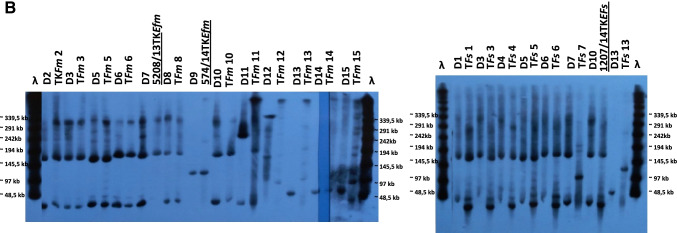
PFGE-S1 Southern blotting results for selected 50 VR*Efs*-VanA and the first Polish VR*EFs*-VanA isolate (7946/1998) (A) and comparative PFGE-S1 Southern blotting with the *vanA* probe for conjugation-positive isolates (donors D1-D15) and their *E. faecalis* and/or *E. faecium* transconjugants (B). Eight isolates analysed by WGS underlined; ^a^ city abbreviations and outbreak designation as in Fig. 2; isolates positive for conjugation in bold

Fifty isolates belonging to both main CCs 2 and 87 (22 and 26 isolates, respectively), as well as representing STs 16 and 215 (single isolates each) obtained from 32 medical centres and representing 21 PFGE types and 14 Tn*1546* types/subtypes, were used in PFGE-S1 Southern-blot hybridization analyses and conjugative transfer experiments (Fig. 3). These isolates constituted 40% of the studied collection and were selected to maximally represent its diversity. The first Polish VR*Efs*-VanA was additionally included in this group. Single *vanA*-plasmids were detected in 13 isolates, while 37 isolates carried two co-resident *vanA*-plasmids, resulting in a total number of 87 *vanA-*plasmids (Fig. 3A).

The most prevalent *repUS1*_pVEF1_-*vanA* plasmids (*n* = 63) were detected in 37 isolates, of which 26 isolates had two *repUS1*_pVEF1_-*vanA* plasmid bands, ≤ 30 kb and 150–200 kb in size (Fig. 3A). The *rel*_pCIZ2_ relaxase gene (MOB_P7_) was associated with the majority of *repUS1*_pVEF1_-*vanA* replicons (50 out of 63 plasmids). The *repUS1*_pVEF1_-*vanA* replicons were observed for outbreak isolates in Warsaw (A, I, D1, D3 and H) as well as in 12 other cities during the whole study period. Isolates with these plasmids carried mainly BB- and B-subtypes of Tn*1546* transposon. *rep2*_pRE25_ was present altogether on 12 *vanA*-replicons, ranging from 20 to 160 kb in size. In six cases, *rep2*_pRE25_ was located on 30–50 kb *repUS1*_pVEF1_-*vanA* plasmids, described above. These putative *rep2*_pRE25_-*repUS1*_pVEF1_-*vanA* multireplicons appeared in 2011 and were associated with two small outbreaks in Wa-8 (D2) and Pl (B) and later they were also detected in Wa-7 and Po-6 sporadic cases in 2016. The *rep1*_pIP501_ gene was associated with four 80–270 kb *vanA*-plasmids, including one 100 kb *rep2*_pRE25_-*vanA* plasmid. The *rep1*_pIP501_-*vanA* plasmids were present among early isolates, mainly from the C outbreak in Po-2 in association with the E-type Tn*1546* transposon.

Other *vanA* replicons were detected sporadically. In a single isolate *rep2*_pRE25_-*repUS1*_pVEF1_-*vanA* plasmid hybridized also with *rep9*_pAD1/pTEF2/pCF10_ gene. Apart from this isolate, *rep9*_pAD1/pTEF2/pCF10_ was also specific for two large *vanA*-plasmids, 300 kb and 360 kb in size. The 360 kb *rep9*_pAD1/pTEF2/pCF10_-*vanA* plasmid carried additionally *rep6*_pS86_. A single isolate carried 80 kb *vanA*-plasmid with both *rep6*_pS86_ and r*ep2*_pRE25_ replication genes. The first Polish VR*Efs*-VanA isolate and the remaining 11 isolates from the collection harboured *vanA-*plasmids ranging from 20 to 270 kb in size; however, their replicon type could not be determined (Fig. 3A).

Fifteen isolates out of 50 selected for Southern-blotting were positive for conjugative transfer of *vanA* determinants to a susceptible recipient (Fig. 3B). Six of them transferred *vanA* plasmids to both *E. faecalis* OG1RF and *E. faecium* 64/3 recipient, seven only to *E. faecium* and two only to *E. faecalis*. The most commonly transferred *vanA* plasmids belonged to *repUS1*_pVEF1_ replicons of ≤ 30 kb and 150–200 kb in size (9 isolates out of 36 isolates with *repUS1*_pVEF1_-*vanA* plasmids investigated, Fig. 3A) and for eight of them, no significant changes in plasmid sizes were observed during conjugation (Fig. 3B). Additionally, two *rep2*_pRE25_-*repUS1*_pVEF1_-*vanA* plasmids (30–50 kb), a single *rep2*_pRE25_-*rep9*_pAD1/pTEF2/pCF10_-*repUS1*_pVEF1_-*vanA* plasmid (50 kb) and a single *rep6*_pS86_-*rep9*_pAD1/pTEF2/pCF10_-*vanA* plasmid (360 kb) were transferable by conjugation, however with significant changes in plasmid size (Fig. 3B). Transfer was also achieved in the case of two isolates harbouring *vanA-*plasmids with unknown replicon types.

To compare the distribution of *rep* genes associated with *vanA-*plasmids in our collection, a set of 22 *vanA-*plasmids from *E. faecalis* reported from other countries was assembled (Supplementary Table 2). The majority of these isolates was obtained from human sources in various countries during 1996–2016. The *vanA-*plasmids in *E. faecalis* varied in size from 31.4 kb to 128 kb and harboured *rep1, rep2, rep7a, rep9b, rep9c, repUS1* and *repUS43,* with one to three *rep* genes per plasmid. Only three plasmids, 31.4, 76.0 and 107.6 kb in size, harboured *repUS1,* characteristic for the majority of *vanA*-plasmids from the current study. The co-localization of *rep18a* with *repUS1*_pVEF1_ on *vanA*-plasmids_,_ common among Polish VR*Efs*-VanA (see below), was not observed elsewhere. Six plasmids in the reference set represented multireplicons with various combinations of *rep* genes. The *rep9*_pAD1/pTEF2/pCF10_-*vanA* plasmids, which were the most common ones in the reference set, were observed only sporadically in our collection (12 vs. 3 plasmids, respectively).

### WGS analysis of selected VREfs-VanA

The complete genome of 1207/14 isolate was reported previously [19] and the Illumina MiSeq sequencing of seven VR*Efs*-VanA isolates and three transconjugants was performed in the current study (Table 2). Selection of isolates for WGS analysis was aimed at providing the best representation of STs, Tn*1546* types and *vanA*-plasmid PFGE-S1 profiles detected in the studied population, including isolates with the most common characteristics as well as those appearing sporadically (Figs. 2 and 3). In particular, WGS was carried out for three isolates harbouring the most prevalent B5-type Tn*1546* in various backgrounds (STs 6, 28, 87), an isolate with the related B9-type in ST16, not belonging to any of the two predominant CCs, an isolate with BC6-type in ST464 (CC87) and single isolates with the A7 and E transposon types associated with the ST6 and ST87, respectively. Additionally, the first Polish VR*Efs*-VanA isolate was also included in WGS.

**Table 2:** Tn*1546*-associated plasmidome of selected VR*Efs* VanA isolates and transconjugants

Strain ID/year of isolation	Tn*1546* flanking regions	Tn*1546* type	Species / TK strain	ST/CC	VanA replicons ^a^	Distribution of 1207/14 plasmids ^b^	Plasmid replicons on *vanA* contigs	Code of medical centre ^c^	NCBI accession no
1207/2014	*rep18a-repUS1*	B5	Efs	464/87	*repUS1*(20), *repUS1*(170)	p1207_1, p1207_2, p1207_3, **p1207_4**, p1207_5, p1207_6	*repUS1, rep18a*	*Ost*	CP075604-CP075610 (19)
1207/2014TK	*rep18a-repUS1*	B5	EfsOG1RF	-	n.d.(20), n.d.(170)	p1207_1, **p1207_4**	*repUS1*	*-*	-
5208/2013	*rep18a-repUS1*	B5	Efs	87/87	*repUS1*(20), *repUS1*(170)	p1207_2, p1207_3, **p1207_4**	*repUS1*	*Wa-13*	JAHDUK000000000
5208/2013TK	*rep18a-repUS1*	B5	Efm64/3	-	n.d.(180)	p1207_2, **p1207_4**	*repUS1*	*-*	-
3124/2008	*rep18a-repUS1*	B5	Efs	28/87	(20), (170)	**p1207_4**	*repUS1*	*Wa-1*	JAHDUH000000000
5274/2012	*ΔrepB-repUS1*	B5	Efs	6/2	*repUS1*(20), *repUS1*(170)	**p1207_4**	*repUS1*	*Wa-2*	JAHDUJ000000000
753/2012	*rep18a-repUS1*	B9	Efs	16/other	*repUS1*(20), *repUS1*(170)	**p1207_4**	*repUS1*	*Wa-2*	JAHDUI000000000
3300/2004	*R-rep1*	E	Efs	87/87	*rep1* (70)	1207_3	*rep1*	*Po-2*	JAHDUG000000000
574/2014	*relE-merA*	A7	Efs	6/2	(120)	p1207_2	none	*My*	JAHDUL000000000
574/2014TK	*relE-merA*	A7	Efm64/3	-	n.d.(120)	p1207_2	none	*-*	-
1739/2015	*R-arsR*	BC6	Efs	464/87	(160), *rep6 rep9* (360)	p1207_1, p1207_2 (75%), p1207_3 (75%)	none	*To-2*	JAHDUM000000000
7946/1998	*R-arsR*	BB3	Efs	87/87	(120)	1207_3 (50%)	none	*Gd-a*	JAHDUF000000000

^a^ S1-PFGE hybridization results, approximate plasmid size in kb in brackets; ^b^ mapping sequencing reads to p1207_1, p1207_2, p1207_3, p1207_4, p1207_5 and p1207_6 plasmids (p1207_4 in bold), with 100% coverage unless indicated otherwise in brackets; ^c^ centre abbreviation according to Fig. 1; n.d., not defined

**Fig. 4:**
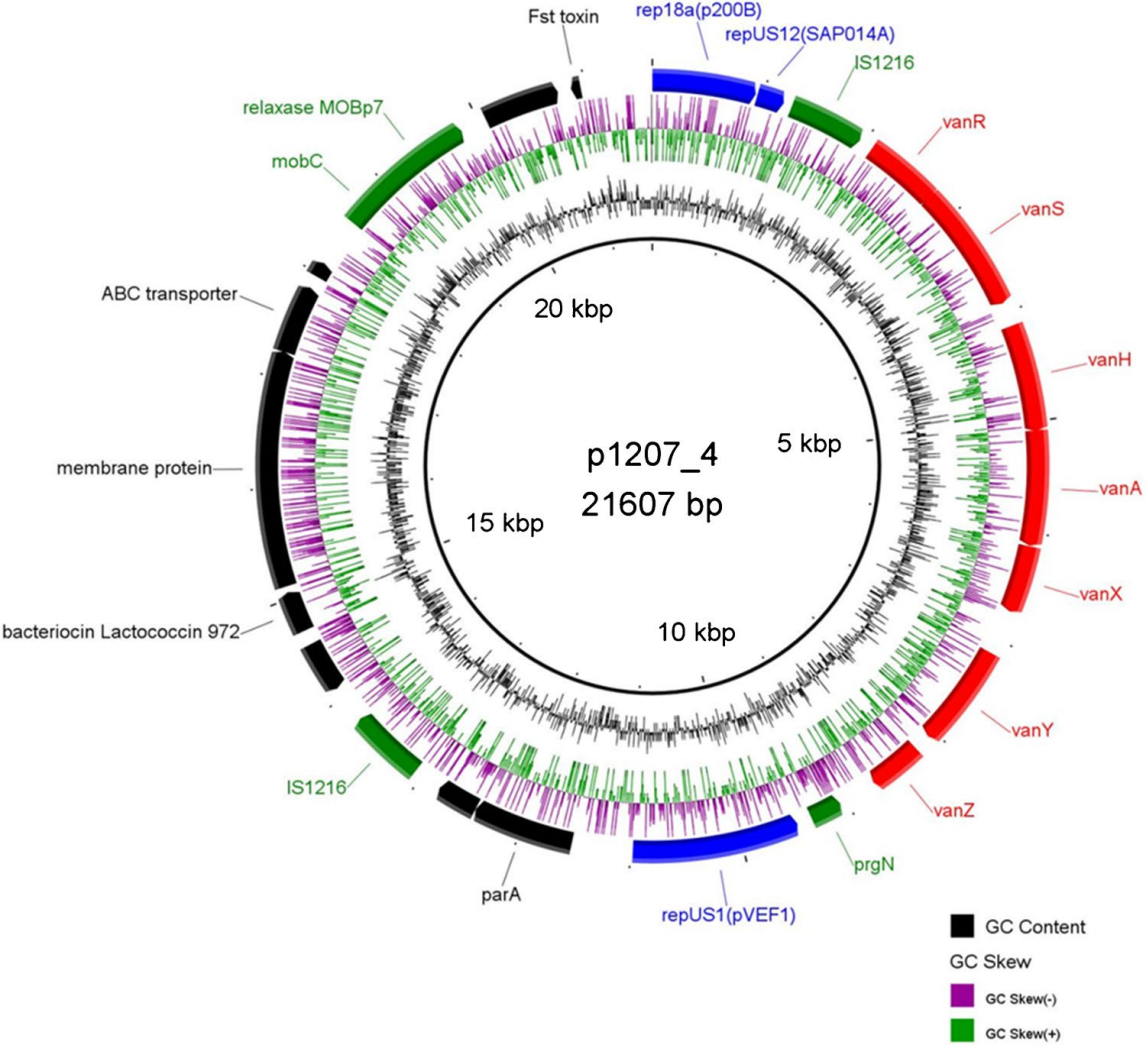
Map of the p1207_4 *vanA*-plasmid (GenBank accession no. CP075608); positions and directions of predicted coding sequences are indicated by arrows, with antimicrobial resistance genes in red, plasmid replication genes in blue, genes associated with conjugative transfer, mobilization and transposition in green and other CDSs in black

The 1207/14 isolate demonstrated the most common ≤ 30 kb and 170 kb *repUS1*_pVEF1_-*vanA* plasmid profile and represented one out of four isolates transferring *vanA* to both *E. faecium* and *E. faecalis* without any noticeable change in size and number of *vanA*-plasmids (donor D10, Fig. 3B). This isolate contained five plasmids (p1207_1-p1207_5, including the 21.6-kb plasmid p1207_4 harbouring the *vanA* operon) with 10 known *rep* genes, and a small 2-kb plasmid with an unknown *rep* type [19]. The analysis in the current study revealed that p1207_4 carries 24 probable protein-coding genes (Fig. 4), including three *rep* genes: (i) *repUS1*_pVEF1_ (Inc18) with 100% identity to the *rep* gene of pVEF1, pVEF2 and pVEF4 plasmids of *E. faecium* [51], (ii) *rep18a*_p200B_ (Rep_3) with 99.98% identity to the *repA* gene of p200B of *E. faecium* [52] and (iii) a truncated *repB* with 100% identity to the *repB* gene present in several plasmids of *E. faecium,* e.g. in the ISMMS and E39 isolates [53, 54]. Screening the p1207_4 sequence for conjugation transfer-associated regions by oriTFinder revealed the presence of a mobilization gene belonging to MOB_P7_ family (*rel*_pCIZ2_); however, no *oriT* was detected. The p1207_4 represented a unique mosaic plasmid structure, composed of two segments separated by IS*1216.* The 8.9 kb segment containing partial *repB, rep18a*_p200B_*, rel, mobC*, the Fst toxin gene and a set of genes, presumably involved in the biosynthesis of a putative bacteriocin belonging to the lactococcin 972 family, demonstrated 99.5% identity to the ISMMS_VRE_p3 plasmid of *E. faecium* [53]. The structure of the other, a 10.7 kb segment, which included a partial Tn*1546* and the *prgN*, *repUS1*_pVEF1_ and *parA* genes was unique in the GenBank.

Illumina MiSeq sequencing of seven VR*Efs*-VanA isolates demonstrated that in four isolates (3124/08, 5274/12, 753/12 and 5208/13) both *vanA* and *repUS1*_pVEF1_ reside on identical contigs, approximately 11 kb in size. As described above, these isolates carried *repUS1-vanA* plasmids of approximately 20 and 170 kb (Fig. 3A), and assembling sequencing reads of these four plasmids to isolate 1207/14 plasmids as reference confirmed the presence of p1207_4 sequences in all four isolates (Table 2). In the case of 5208/13, mapping Illumina MiSeq reads revealed the presence of the complete sequences of p1207_2 and p1207_3 in addition to p1207_4 (Table 2). The p1207_2 plasmid (70.1 kb) is an Inc18-plasmid, harbouring *repUS11*_pTEF3_ and conjugative transfer functions, while p1207_3 is a 46.5-kb plasmid with *rep9a*_pAD1_ [19]. A single isolate (3300/04) exhibited the presence of approximately 20 kb *vanA/rep1*_pIP501_ contig, consistent with the previous Southern blotting results (Fig. 3A), and mapping Illumina reads of 3300/04 revealed the presence of complete sequences of p1207_3 (Table 2). The *vanA*-contigs of three remaining isolates (7946/98, 574/14 and 1739/15) contained no plasmid replication genes (Table 2). Mapping Illumina reads of 574/14 and 1739/15 isolates revealed the presence of p1207_2 and p1207_1 plasmid sequences in these isolates, respectively.

Three transconjugants, 1207/14TK*Efs*, 5208/13TK*Efm* and 574/14TK*Efm*, were selected for Illumina MiSeq sequencing. Mapping sequencing reads of 1207/14TK*Efs* to the plasmid sequences from the 1207/14 donor confirmed the presence of the p1207_4 plasmid in 1207/14TK*Efs* and additionally revealed acquisition of the p1207_1 plasmid by this transconjugant. p1207_1 is the largest plasmid found in 1207/14 (75.1 kb) and carries four *rep* genes (*rep9b*_pEF62pC_, *rep6*_pS86_, *rep7a*_pRE25_ and *repUS12*_SAP014A_), conjugation transfer genes and antimicrobial resistance determinants *aph(3')-III, ant(6)-Ia, erm(B)* and *cat* [19]. In the case of 5208/13 transconjugants, the change of the original *repUS1*_pVEF1_-*vanA* plasmid profile (20 kb and 170 kb) was observed (a single 170 kb *repUS1*_pVEF1_-*vanA* plasmid in 5208/13TK*Efm* and a single 110 kb *repUS1*_pVEF1_-*vanA* plasmid in 5208/13TK*Efs*, respectively, as shown in Fig. 3B). Mapping Illumina MiSeq reads revealed the presence of the complete sequences of p1207_2 in addition to p1207_4 in 5208/13TK*Efm* transconjugant (Table 2). For the 574/14 isolate transfer of *vanA* to *E. faecalis* OG1RF was not achieved, while the 574/14TK*Efm* transconjugant carried a single 120 kb *vanA*-plasmid, identical to the *vanA*-plasmid band observed for the donor (Fig. 3B). Mapping Illumina reads revealed the presence of complete p1207_2 sequence in 574/14TK*Efm* (Table 2). Plasmid replication genes typical for p1207_1, p1207_2 and p1207_4 were the only *rep* genes present in the analysed transconjugants, according to the PlasmidFinder results.

### Relationships of CC87 and ST6 isolates with isolates from other countries

Genomic sequences of six Polish isolates belonging to CC87 and two Polish isolates of ST6 were used for joint analyses with genomic sequences available at GenBank. The reference set for CC87 consisted of 23 isolates, originating from 12 other countries and obtained during 1986–2020 from various sources (Supplementary Fig. 1A). The Roary analysis identified 6203 genes, of which 2295 represented the core genome. In the ML tree (Supplementary Fig. 1A), three Polish ST87 isolates, including the first Polish VR*Efs*-VanA from 1998 and two isolates from 2004 and 2013, formed a group separated from other Polish CC87 isolates. Instead, these three isolates clustered with the majority of ST28 isolates from other countries. Although the three isolates appeared very closely related in the core-genome analysis, each of them had a unique content of plasmid *rep* genes and resistance determinants, and carried the Tn*1546-*type transposon in a different genetic context (see below), suggesting an independent acquisition of the *vanA* determinant. Two Polish isolates of ST464 and a single Polish ST28 isolate formed two separate branches, unique for Poland. Two ST464 isolates were very closely related in the core-genome analysis and shared antimicrobial resistance determinants but demonstrated a different genetic context of Tn*1546* (see below). For the analysis of ST6 isolates, 155 genomic sequences were retrieved from GenBank. To improve the clarity of presentation, very closely related or indistinguishable isolates were removed from an initial ML tree (data not shown) and such clusters were represented by a single randomly chosen isolate (Supplementary Fig. 1B). The final ML tree included 33 reference sequences, characteristic for human isolates obtained during 1986–2019 from 10 countries. The pangenome in this group consisted of 6375 genes, of which 2048 belonged to the core genome. Two Polish isolates were closely related in this analysis and belonged to a larger cluster, grouping several vancomycin-resistant isolates from the USA from the second half of the 2010s as well as single isolates from the UK and Denmark. Both CC87 and ST6 isolates demonstrated the abundance of resistance determinants (up to 8 and 7 genes per isolate in CC87 and ST6 groups, respectively) and plasmid *rep* genes (up to 8 genes per isolate in both groups). The most ubiquitous genes included *tet*(M), *erm*(B) and aminoglycoside high-level resistance genes as well as *rep9a* and *rep9b*, characteristic for pheromone-responsive plasmids. The *rep18a*_p200B_ and *repUS1*_pVEF1_ genes flanking Tn*1546* in the majority of isolates in our collection (see below) were observed solely for three Polish isolates from CC87 and a single Polish isolate of ST6. No particular associations between the distribution of resistance genes or *rep* genes with the ML tree groupings were found.

### The genetic context of Tn1546

The analysis of Tn*1546* flanking regions on p1207_4 plasmid [19] revealed that *rep18a*_p200B_ and partial *repB* genes were present upstream and the *prgN* and *repUS1*_pVEF1_ genes were located downstream of the transposon*.* This type of Tn*1546* flanks was designated *rep18a-repUS1* and was not as yet observed elsewhere (GenBank nr/nt database, 23^rd^ June 2022, date last accessed). The same genetic neighbourhood downstream Tn*1546* was found also in *vanA*/*repUS1*_pVEF1_ contigs of four isolates analysed by WGS (see above)*.* However, regions upstream Tn*1546* were lacking from these contigs, consistent with the presence of IS*1216* in B5- and B9-type transposons in these isolates, which precluded assembly of this region from Illumina MiSeq reads. The investigation of Tn*1546* insertion sites in the remaining four isolates analysed with the Illumina MiSeq sequencing allowed to define the following Tn*1546* flanks: (i) R-*rep1* with the *rep1*_pIP501_, *res* and *topoI* genes downstream of Tn*1546*; (ii) *relE-merA* with the *relE* toxin gene upstream of Tn*1546* and the *merA* gene downstream of Tn*1546* and (iii) R-*arsR* with the *arsR* gene downstream of transposon (Fig. 1, Table 2). The R-*rep1* flank was not reported previously. The flank designated *relE-merA* was described recently in three *vanA* plasmids (pVB096, pVB039 and pVBR48) from *E. faecium* ST133 isolates [55]. The R-*arsR* flanking sequence, typical for the first Polish VR*Efs-*VanA isolate, was commonly detected in *E. faecium* genomes (116 hits in GenBank) while only a single *E. faecalis* plasmid pR712_01 with an identical flank was deposited in GenBank (CP036247.1).

Based on the sequences of four types of flanks described above, PCR primers specific to junctions between plasmids and Tn*1546* were designed (Supplementary Table 1) and used for screening the whole collection (Figs. 1 and 2). A hundred-nine isolates (87.2%) had the *repUS1* downstream flank but only 92 of them yielded the PCR product of the expected size for the *rep18a* upstream flank. Four of the remaining isolates yielded smaller PCR products due to either deletion within partial *repB* (3 isolates, the flank designated *ΔrepB-repUS1*) or deletion encompassing a fragment of *rep18a*_p200B_ and almost entire *repB* gene (1 isolate, the flank designated Δ[*rep18a*-*repB*]*-repUS1*). For seven isolates obtained from various Warsaw medical centres during 2014–2016, the upstream flanking region was amplified with the use of a new primer rep18a_up_new, located ~ 500 bp upstream of the previously used primer rep18a_up, revealing the presence of IS*Efa4* insertion within *rep18a*_p200B_ (the flank designated *ISEfa4-repUS1*). For seven remaining isolates, the left flank could not be defined and these flanking sequences were designated *R-repUS1*. Among the remaining 17 isolates negative for the *rep18a-repUS1* flanks, eight outbreak isolates from the Po-2 medical centre in 2004 (Tn*1546* type E) showed the presence of *R-rep1*, four unrelated isolates carried *R-arsR*, while *relE-merA* remained typical only for the 574/2014 isolate, analysed previously by WGS. In the case of four remaining unrelated isolates, the Tn*1546* insertion site could not be identified.

Distribution of different Tn*1546* flanking regions in the studied population highly correlated with the results of Tn*1546* typing (Figs. 1 and 2). Isolates with the *rep18a-repUS1* flanks were prevalent during the entire study period and these flanks were associated mainly with the common B5, B4 and BB4 subtypes of Tn*1546*. *ISEfa4-repUS1* flank, associated with B5 and B4 subtypes, was detected during the Wa-12/E outbreak and among sporadic isolates in two other Warsaw medical centres during 2014–2016. The *R-rep1* flanking sequence was typical for E-type transposon in the C outbreak isolates from Po-2. The *R-arsR* flanking region, besides of being typical for the first VR*Efs*-VanA isolate, appeared sporadically in 2015–2016 in association with BC6 and BBJ transposon types. Thirty-one of 39 isolates carrying *repUS1*_pVEF1_ downstream Tn*1546* demonstrated the characteristic plasmid profile with ≤ 30 kb and 150–200 kb *vanA* plasmids in PFGE-S1/hybridization with the *repUS1* probe (Fig. 3).

## Discussion

The results obtained in this study allowed identifying two clonal complexes, CC2 and CC87, as the ones being mostly responsible for an increasing prevalence of VR*Efs*-VanA in Poland. Early studies showed that both these CCs were characterized by multi-drug resistance and an increased pathogenicity potential [4, 5, 25]. These findings were further supported by genomic analyses of 168 isolates of *E. faecalis* from the UK, among which three major lineages L1 (corresponding to CC2), L2 (corresponding to CC87) and L3 demonstrated a strong enrichment in several virulence and antimicrobial resistance genes, including *van* genes [17]. CC2 is dispersed globally, while CC87 is observed in Europe (including Poland), Asia and Africa [3–5, 56; https://pubmlst.org/organisms/enterococcus-faecalis, 27^th^ October 2021 date last accessed]. In the current analysis, we noticed a significant replacement of CC87 by CC2 after 2010 and a similar time-trend in the distribution of the L1 and L2 lineages of *E. faecalis* occurred in British hospitals, where CC87/L2 practically disappeared after 2006 [17]. Both CC2/L1 and CC87/L2 include vancomycin-susceptible as well as vancomycin-resistant isolates [3, 5, 17], consistent with multiple acquisitions of *van* determinants by strains of these CCs.

Tn*1546* transposon is a basic genetic element responsible for *vanA* distribution in enterococcal populations and its genetic diversity serves as epidemiological marker to trace horizontal transfer of glycopeptides resistance among strains and dissemination of VRE-VanA within and among medical centres [11, 45, 57]. However, in contrast to the situation in *E. faecium,* the diversity of Tn*1546* in *E. faecalis* remains much less studied. Among Polish VR*Efs,* IS*1216* seems to play a major role in shaping the Tn*1546* structure*.* This IS was responsible for the formation of B-type transposons most commonly detected during the entire study period and insertion of IS*1216* was associated with deletions of the Tn*1546* left end. Mapping genomic sequencing reads to Tn*1546* as a reference revealed several examples of similar structures of Tn*1546,* i.e. devoid of the transposase and resolvase genes, among all three main lineages of *E. faecalis* in the UK [17]. In the set of *vanA*-plasmids assembled from the GenBank in the current study (Supplementary Table 2), the majority of the observed Tn*1546* structures (13 out of 22) represented the wild-type Tn*1546.* Variants similar to the B-type transposons found in our study were also present in human isolates from Brazil and Portugal. Insertion of IS*1216* between *vanX* and *vanY,* characteristic for the B8, BB3, BB4, BB6, BJ and BBJ types in our collection occurred also in Tn*1546* located on plasmids of *E. faecalis* from human and chicken samples from Korea [58]. Apart from IS*1216,* three other ISs, IS*Efa4*, IS*1251* and IS*1542* were sporadically detected within Tn*1546* in our collection*.* These ISs did not occur in Tn*1546* of *E. faecalis* from other countries; however, some other ISs, such as IS*3*, IS*256* and IS*1657*, were found. B4- and B5-type transposons commonly observed in our study were also present among *E. faecium*-VanA from Polish hospitals (31 and unpublished results), suggesting an extensive genetic exchange of the *vanA* mobilome between *E. faecalis* and *E. faecium*. Moreover, the E-type Tn*1546* with IS*Efa4* characterized in the current study in *E. faecalis* occurred concomitantly in *E. faecium* in the same hospital Po-2, in both cases associated with the *rep1* replicon [31] additionally supporting the possibility of exchange of this element among enterococci in the hospital settings, with a broad host range Inc18 replicon as its vector. Such interspecies transfer was already suggested for the first VRE outbreak in Poland in 1998 [14].

Tn*1546-*type transposons are usually carried on conjugative plasmids from the Inc18 and RepA_N families, which promote their horizontal transfer in bacterial populations [10, 15, 16, 59–61]. In the current study, the majority of *vanA-*replicons belonged to the Inc18 family, being associated mainly with *repUS1* and occasionally with *rep1* and *rep2* while pheromone-responsive RepA_N plasmids played a much lesser role as *vanA* carriers*.* In our PFGE-S1-hybridization studies, the *repUS1* gene was typically associated with ≤ 30 kb and 150–200 kb *vanA-*plasmids in the majority of isolates. Such a profile was observed in several PFGE types of both epidemic CCs 2 and 87 as well as in other STs such as 16 and 215, consistent with independent acquisitions of the *vanA*-plasmid. In the set of 22 reference VR*Efs vanA*-plasmids (Supplementary Table 2), the majority was below 100 kb in size. The *repUS1* replicons were observed in two animal isolates and single human isolate from diverse localizations (The Netherlands, New Zealand, Brazil); one of these plasmids harboured also *rep9c*. The *repUS1*_pVEF1_ gene was first described in pVEF1 and pVEF2 of *E. faecium* from human and poultry in Norway [51]. Although p1207_4, similarly to pVEF1 and pVEF2 lacked a conjugation system, it demonstrated the presence of a relaxase gene and could be mobilized, presumably by conjugative plasmids present in the same bacterial cell, such as a pheromone responsive plasmid p1207_1, co-transferred to the *E. faecalis* transconjugant of the 1207/14 isolate (Table 2). Formation of larger cointegrates, likely with an involvement of very ubiquitous IS*1216*, might explain the existence of larger, approximately 170 kb plasmids hybridizing with the *vanA* probe, apart from the main 21.6 kb form of the *vanA-*plasmid. Formation of such fusions was indeed demonstrated for larger conjugative plasmids and smaller mobilizable *poxtA-*plasmids, enabling transfer of linezolid resistance from *E. faecalis* and *Enterococcus lactis* [62]. The majority of *repUS1*_pVEF1_-*vanA* plasmids represented multireplicons, which contained also *rep18a*, located upstream Tn*1546* and in some instances *repUS1*_pVEF1_-*vanA* plasmids carried also *rep1, rep2* and/or *rep9*. Multireplicons with various combinations of *rep* genes were present also in our plasmid reference set and in some instances such plasmids might represent more stable fusions formed during conjugation, described above. However, it is worth emphasizing that the presence of plasmids of very similar sizes, indistinguishable by PFGE-S1, may provide an alternative explanation for detecting multireplicons by hybridization in some isolates.

In conclusion, in Poland VanA-*E. faecalis* represents a heterogonous group of isolates that emerged from the general population of this species through several acquisitions of *vanA* determinants. The broad-host range mobilizable *repUS1*_pVEF1_-Inc18 replicons were the most common carriers of Tn*1546* and these plasmids played an important role in the dissemination of VanA glycopeptide resistance among *E. faecalis.* Association of *vanA* with specific genetic background, i.e. mostly with multiresistant CCs 2 and 87, contributed to efficient spread of VanA-*E. faecalis* in and among hospitals in Poland, constituting a significant threat to public health.

## Supplementary Information

Below is the link to the electronic supplementary material.

Supplementary file 1 Supplementary Fig. 1. Genome-based relationships among CC87 (A) and ST6 (B) isolates from Poland and other countries. The ML tree (left side of the figure) was constructed with RAxML based on core genome alignment, generated in Roary. Twenty-three genomic sequences of isolates belonging to CC87 and 33 representatives of ST6 were downloaded from GenBank (23^rd^ February 2022) and supplemented with available data on isolation country, source and year (right side of the figure). Two-letter country code follows the international standard ISO3166-1 alpha-2 (https://www.iso.org/iso-3166-country-codes.html; 22^nd^ March 2022 date last accessed). Y, presence; 0, absence; n/a, data not available; data for isolates from the current study in bold. In the tree constructed for ST6 (B), isolates represented by a single representative isolate (underlined) are provided within brackets as follows: 1248_EFLS (1308_EFLS), 132_EFLS (133_EFLS), 1333_EFLS (1325_EFLS), V583 (V587, NCTC13379), B1586 (B1005, B1290, B1376, B4148, B878, B939, B1327, B1696, B1719, B2593, B2867, B3053, B4267, B4259, B4568, B4672, B4411, B1851, B1138, B5076, B1249, B1843, B2391, B2813, B3126, B4008, B4674, B2670, B4018, B5035, B1623, B1933, B2535, B2557, B1532, B1734, B2949, B1505, B1678, B1874, B2202, B2255, B2211, B2277, B2488, B2687, B2685, B2864, B3042, B3286, B4163, B4638, B4969, B3031, B3119, B3196, B2802, EnGen0427), SF100 (SF19), VRE32631 (VRE33430, VRE33236, VRE33319, VRE33481, VRE34517, VRE34684, VRE33143, VRE33492, VRE33271, VRE33454, VRE32839, VRE33670), VRE32867 (VRE32870), VRE32924 (VRE32954, VRE33211), VRE32879 (VRE33353, VRE33801, VRE32930, VRE33535, VRE34808, VRE33107, VRE33257, VRE33766).(PPTX 389 KB)

Supplementary file2 (DOCX 27 KB)

Supplementary file3 (DOCX 23 KB)

## Data Availability

The datasets generated during and/or analysed during the current study are available in The National Center for Biotechnology Information (NCBI) repository, https://www.ncbi.nlm.nih.gov/bioproject/PRJNA731638. The accession numbers of genomic sequences of particular isolates are provided in Table 2.
