# Supplementary figures and images for: VanA-Enterococcus faecalis in Poland: hospital population clonal structure and vanA mobilome
Source: Eur J Clin Microbiol Infect Dis. 2022 Sep 3;41(10):1245–61. doi: 10.1007/s10096-022-04479-4 (PMC9489580; doi:10.1007/s10096-022-04479-4)

## Slide 1
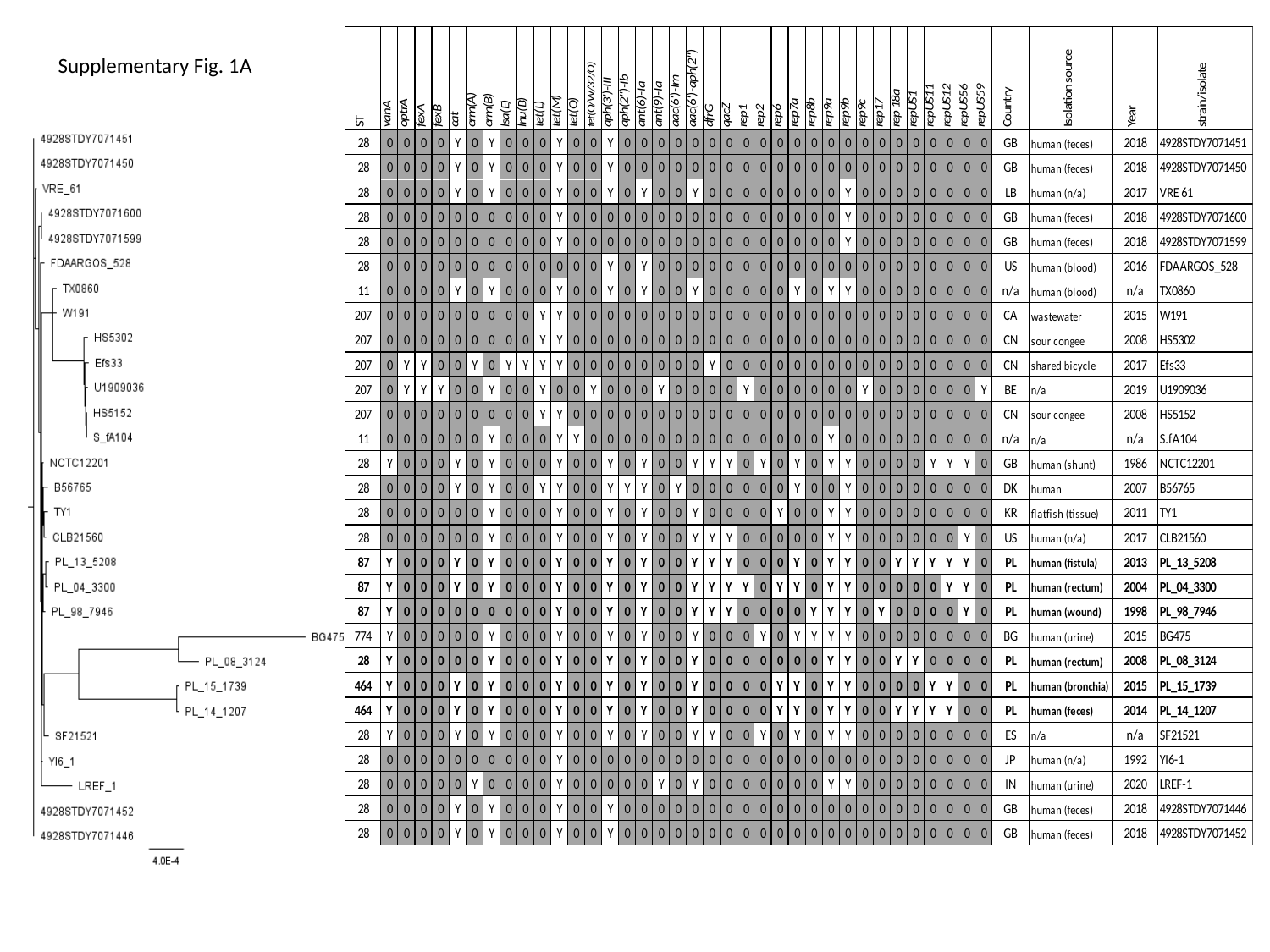

Supplementary Fig. 1A

## Slide 2
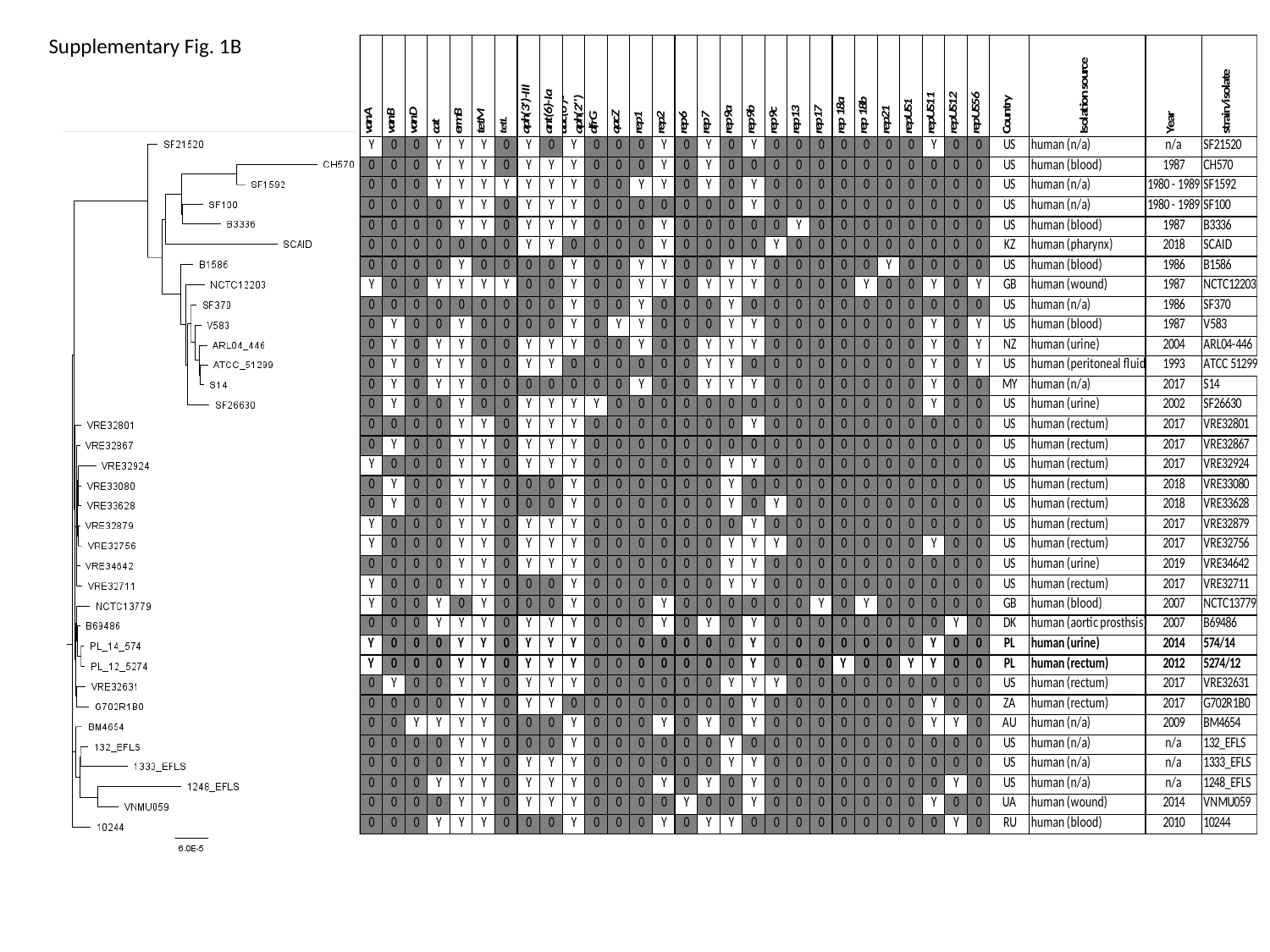

Supplementary Fig. 1B

Supplement: Supplementary file 1 — Supplementary Fig. 1. Genome-based relationships among CC87 (A) and ST6 (B) isolates from Poland and other countries. The ML tree (left side of the figure) was constructed with RAxML based on core genome alignment, generated in Roary. Twenty-three genomic sequences of isolates belonging to CC87 and 33 representatives of ST6 were downloaded from GenBank (23rd February 2022) and supplemented with available data on isolation country, source and year (right side of the figure). Two-letter country code follows the international standard ISO3166-1 alpha-2 (https://www.iso.org/iso-3166-country-codes.html; 22nd March 2022 date last accessed). Y, presence; 0, absence; n/a, data not available; data for isolates from the current study in bold. In the tree constructed for ST6 (B), isolates represented by a single representative isolate (underlined) are provided within brackets as follows: 1248_EFLS (1308_EFLS), 132_EFLS (133_EFLS), 1333_EFLS (1325_EFLS), V583 (V587, NCTC13379), B1586 (B1005, B1290, B1376, B4148, B878, B939, B1327, B1696, B1719, B2593, B2867, B3053, B4267, B4259, B4568, B4672, B4411, B1851, B1138, B5076, B1249, B1843, B2391, B2813, B3126, B4008, B4674, B2670, B4018, B5035, B1623, B1933, B2535, B2557, B1532, B1734, B2949, B1505, B1678, B1874, B2202, B2255, B2211, B2277, B2488, B2687, B2685, B2864, B3042, B3286, B4163, B4638, B4969, B3031, B3119, B3196, B2802, EnGen0427), SF100 (SF19), VRE32631 (VRE33430, VRE33236, VRE33319, VRE33481, VRE34517, VRE34684, VRE33143, VRE33492, VRE33271, VRE33454, VRE32839, VRE33670), VRE32867 (VRE32870), VRE32924 (VRE32954, VRE33211), VRE32879 (VRE33353, VRE33801, VRE32930, VRE33535, VRE34808, VRE33107, VRE33257, VRE33766).(PPTX 389 KB) [file 10096_2022_4479_MOESM1_ESM.pptx]
