# Supplementary material for: VanA-Enterococcus faecalis in Poland: hospital population clonal structure and vanA mobilome
Source: Eur J Clin Microbiol Infect Dis. 2022 Sep 3;41(10):1245–61. doi: 10.1007/s10096-022-04479-4 (PMC9489580; doi:10.1007/s10096-022-04479-4)
Supplement: Supplementary file 2 — Supplementary file2 (DOCX 27 KB) [file 10096_2022_4479_MOESM2_ESM.docx]

VanA-*Enterococcus faecalis* in Poland: long-term dynamics of hospital population and mobilome

European Journal of Clinical Microbiology & Infectious Diseases

Ewa Wardal, Dorota Żabicka, Waleria Hryniewicz and Ewa Sadowy

Dr Ewa Sadowy

Department of Molecular Microbiology, National Medicines Institute, Warsaw, Poland

E-mail address: [e.sadowy@nil.gov.pl](mailto:e.sadowy@nil.gov.pl)

**Supplementary Table 1.** Primers used for Tn*1546* typing and detection of plasmid addiction systems and relaxase genes.

| **Tn*1546* typing and defining Tn*1546* flanking regions** | | | | | | | |
| --- | --- | --- | --- | --- | --- | --- | --- |
| Primer names | Sequence (5’-3’) | | Position in reference sequence | | Reference sequence | | Literature |
| tn1546-1 | GGAAAATGCGGATTTACAACGCTAAG | | 13-38 | | M97297 | | 1 |
| ORF1_4 | GCATGTAGTGATGAAACACCTAGCTGC | | 949-975 | | M97297 | | 2 |
| ORF1_5 | CACGTCCTGCCGACTATGATTATTT | | 1915-1891 | | M97297 | | 2 |
| ORF2_F | TCATTCCATTTCTGTATTTTCAATTT | | 3048-3073 | | M97297 | | 3 |
| ORF2_R | GCCCATTAGCGGAATACAGA | | 3786-3767 | | M97297 | | 3 |
| ORF2_F2 | ACTAATGTATCTAGGGCTTCA | | 3709-3729 | | M97297 | | 3 |
| vanR_R | GCAATTTCATGTTCATCATCCA | | 4019-3998 | | M97297 | | 3 |
| vanS_R | GCTGGAAGCTCTACCCTAAA | | 5769-5750 | | M97297 | | 3 |
| vanS | AACGACTATTCCAAACTAGAA | | 4676-4696 | | M97297 | | 2 |
| vanH2 | GAGCATGGAATGCATCTGCC | | 6081-6062 | | M97297 | | 4 |
| vanS1 | ATTGTTCAGCATGGAGGGC | | 5696-5714 | | M97297 | | 4 |
| vanA1 | CATGAATAGAATAAAAGTTGCAATA | | 6978-7002 | | M97297 | | 5 |
| vanA2 | CCCCTTTAACGCTAATACGATCAA | | 8007-7984 | | M97297 | | 5 |
| vanX2 | TTATTTAACGGGGAAATC | | 8624-8607 | | M97297 | | 6 |
| vanX1 | ATGGAAATAGGATTTACTT | | 8016-8034 | | M97297 | | 6 |
| vanY1 | AGAGACGAACCATACCCCAA | | 8577-8596 | | M97297 | | 3 |
| vanX_F | ATGGGTATTTTCAGAAGTCCC | | 9213-9193 | | M97297 | | 3 |
| vanY2-R | AGTATGTGTTGATCCGGGAAAC | | 9948-9927 | | M97297 | | 7 |
| vanZ1 | CTGGGAATTTCAGAGAGATG | | 10258-10277 | | M97297 | | 4 |
| vanZ2 | AATGGGTACGGTAAACGAGC | | 10581-10562 | | M97297 | | 4 |
| tn1546-2 | GGAAAATGCGGATTTACAACGTTAAG | | 10839-10814 | | M97297 | | 1 |
| IS1216F | CCGTGGGCTACTATCTTCGTT | | 124-144 | | U49512 | | 8 |
| IS1216R | AATTTATTGCGTCTCTTTACTGGA | | 610-587 | | U49512 | | 8 |
| repUS1_dn(2) | CAATAAGATCGGCGTGCAAAC | | nd | | AM296544 | | ^this study^ |
| rep1_dn | CAAAACGGACACACAACTCG | | nd | | U83488 | | ^this study^ |
| 7946_dn | TCGCTACAACGTCACACCAT | | nd | | 7946/98 MiSeq | | ^this study^ |
| merA_dn | TATTGAACTTGAGCGCAGCAG | | nd | | 574/14 MiSeq | | ^this study^ |
| vanR_dn | TTAACTCCAGTGGGCGAAAG | | nd | | M97297 | | ^this study^ |
| rep18a_up_new | GTTTCGGAAGAAAAAGCAATGGT | | nd | | AB158402 | | ^this study^ |
| vanZ_A_up | GCTCGTTTACCGTACCCATT | | nd | | M97297 | | ^this study^ |
| **Plasmid-associated genes** | | | | | | | |
| Primer names | | Sequence (5’-3’) | | Length of amplified PCR fragment in bp | | Literature | |
| rel-pAD1-1 | | TAATTTATGTRACACCTAGT | | 133 | | 9 | |
| rel-pAD1-2 | | TCAAATTCTTCRGCTGAAAAAAYATGC | |  |  |  |  |
| rel-pRE25-1 | | AATGGCTTCTTATCGAAGTGG | | 325 | | 9 | |
| rel-pRE25-2 | | TTCATCATCTCTATGAATGG | |  |  |  |  |
| rel-pCF10-1 | | TAATCCAATCMTTTTCTC C | | 318 | | 9 | |
| rel-pCF10-2 | | TGGACTSWGTTTGCCATTTTG | |  |  |  |  |
| rel-pCIZ2-1 | | ATGGCAACAGTTAAAGTAAG | | 208 | | 9 | |
| rel-pCIZ2-2 | | CAGCGGTAATTTCTCCTGGTC | |  |  |  |  |
| rel-Tn1549-1 | | GTAAGGGCAAGAGCTATGCC | | 265 | | 9 | |
| rel-Tn1549-2 | | ACGCGGCCTTTGATACGCTC | |  |  |  |  |
| rel-BM4518-1 | | GCAAGAAAAAGCCCTATGCC | | 278 | | 9 | |
| rel-BM4518-2 | | GGAATTTTCGCTGATACGCTC | |  |  |  |  |
| rel-pEF1071-1 | | CCATGGCCGAACGAAAACTC | | 252 | | 9 | |
| rel-pEF1071-2 | | ACTCATGTTTAATCACGTCC | |  |  |  |  |
| rel-pAMa1.E-1 | | GAAAGACACAAATTTAAGTGG | | 375 | | 9 | |
| rel-pAMa1.E-2 | | ACAATTCCTAAGTGCATATGG | |  |  |  |  |
| e-z-F | | GTGGTTTAGGTGGCTGCAAG | | 1044 | | 10 | |
| e-z-R | | TTAACGAATTATCGGCAAGC | |  |  |  |  |
| par-F | | CCATGCACTACTAGGCAACC | | 747 | | 10 | |
| par-R | | CTGTCTAGCAAGCAGAGTTACG | |  |  |  |  |
| efm-repA_pLG1-up | | GAAAATGATATCTACTTACTCG | | 568 | | 7 | |
| efm-repA_pLG1-dn | | TTACATAGACAAAAATCAGGT | |  |  |  |  |
| Efs_bee1_up | | GAACTCGATTCTGATACGAATGTCTTTAA | | 259 | | 11 | |
| Efs_bee1_dn | | GTCGAATGCCTTGTCCACCAGTAGATGGC | |  |  |  |  |
| Efs_bee2_up | | CGGACAAAGAATTAGCTACCGTTCAC | | 532 | | 11 | |
| Efs_bee3_dn | | CAAGATAAGCATCTGTTACATCATAGGCTG | |  |  |  |  |
| E-cytI(cyt) | | ACTCGGGGATTGATAGGC | | 688 | | 12 | |
| E-cytIib | | GCTGCTAAAGCTGCGCTT | |  |  |  |  |
| Asa_cons_700-up | | GAAAATGCAGAAATTGCRGC | | 1040-1073 | | 13 | |
| Asa_cons_1700-dn | | GGAACAAGAACAGGAACSAC | |  |  |  |  |
| repUS1_up | | TCGACGGTATTACAAAATGG | | 242 | | ^this study^ | |
| repUS1_dn | | ATTCTGGCTTCATCAGCATA | |  |  |  |  |
| rep18a_up | | AAATGGCGTGCTAAAGATCGAAT | | 330 | | ^this study^ | |
| rep18a_dn | | TTCAGCCCATCCTTGAGTTC | |  |  |  |  |

References

1. Palepou MF, Adebiyi AM, Tremlett CH, Jensen LB, Woodford N (1998) Molecular analysis of diverse elements mediating VanA glycopeptide resistance in enterococci. J Antimicrob Chemother 42(5):605-12. doi: 10.1093/jac/42.5.605.
2. Huh JY, Lee WG, Lee K, Shin WS, Yoo JH (2004) Distribution of insertion sequences associated with Tn1546-like elements among *Enterococcus faecium* isolates from patients in Korea. J Clin Microbiol 42(5):1897-902. doi: 10.1128/JCM.42.5.1897-1902.2004..
3. Talebi M, Pourshafie MR, Katouli M, Möllby R (2008) Molecular structure and transferability of Tn1546-like elements in *Enterococcus faecium* isolates from clinical, sewage, and surface water samples in Iran. Appl Environ Microbiol 74(5):1350-6. doi: 10.1128/AEM.02254-07.
4. Jensen LB, Ahrens P, Dons L, Jones RN, Hammerum AM, Aarestrup FM (1998) Molecular analysis of Tn*1546* in *Enterococcus faecium* isolated from animals and humans. J Clin Microbiol 36(2):437-42. doi: 10.1128/JCM.36.2.437-442.1998.
5. Clark NC, Cooksey RC, Hill BC, Swenson JM, Tenover FC (1993) Characterization of glycopeptide-resistant enterococci from U.S. hospitals. Antimicrob Agents Chemother 37(11):2311-7. doi: 10.1128/AAC.37.11.2311.
6. Yu HS, Seol SY, Cho DT (2003) Diversity of Tn*1546*-like elements in vancomycin-resistant enterococci isolated from humans and poultry in Korea. J Clin Microbiol 41(6):2641-3. doi: 10.1128/JCM.41.6.2641-2643.2003.
7. Wardal E, Markowska K, Zabicka D, Wróblewska M, Giemza M, Mik E, Połowniak-Pracka H, Woźniak A, Hryniewicz W, Sadowy E (2014) Molecular analysis of *vanA* outbreak of *Enterococcus faecium* in two Warsaw hospitals: the importance of mobile genetic elements. Biomed Res Int 2014:575367. doi: 10.1155/2014/575367.
8. Tsai JC, Hsueh PR, Chen HJ, Tseng SP, Chen PY, Teng LJ (2005) The *erm(T)* gene is flanked by IS*1216V* in inducible erythromycin-resistant *Streptococcus gallolyticus* subsp. *pasteurianus*. Antimicrob Agents Chemother 49(10):4347-50. doi: 10.1128/AAC.49.10.4347-4350.2005.
9. Freitas AR (2011) Ecology and evolution of antimicrobial resistance in *Enterococcus*: A multilayered molecular approach with emphasis in plasmid diversity. PhD Thesis
10. Moritz EM, Hergenrother PJ (2007) Toxin-antitoxin systems are ubiquitous and plasmid-encoded in vancomycin-resistant enterococci. Proc Natl Acad Sci U S A 104(1):311-6. doi: 10.1073/pnas.0601168104.
11. Tendolkar PM, Baghdayan AS, Shankar N (2006) Putative surface proteins encoded within a novel transferable locus confer a high-biofilm phenotype to *Enterococcus faecalis*. J Bacteriol 188(6):2063-72. doi: 10.1128/JB.188.6.2063-2072.2006..
12. Vankerckhoven V, Van Autgaerden T, Vael C, Lammens C, Chapelle S, Rossi R, Jabes D, Goossens H (2004) Development of a multiplex PCR for the detection of *asa1*, *gelE*, *cylA*, *esp*, and *hy*l genes in enterococci and survey for virulence determinants among European hospital isolates of *Enterococcus faecium*. J Clin Microbiol 42(10):4473-9. doi: 10.1128/JCM.42.10.4473-4479.2004.
13. Wardal E, Gawryszewska I, Hryniewicz W, Sadowy E (2013) Abundance and diversity of plasmid-associated genes among clinical isolates of *Enterococcus faecalis*. Plasmid 70(3):329-42. doi: 10.1016/j.plasmid.2013.07.003.
