# Supplementary material for: VanA-Enterococcus faecalis in Poland: hospital population clonal structure and vanA mobilome
Source: Eur J Clin Microbiol Infect Dis. 2022 Sep 3;41(10):1245–61. doi: 10.1007/s10096-022-04479-4 (PMC9489580; doi:10.1007/s10096-022-04479-4)
Supplement: Supplementary file 3 — Supplementary file3 (DOCX 23 KB) [file 10096_2022_4479_MOESM3_ESM.docx]

VanA-*Enterococcus faecalis* in Poland: hospital population clonal structure and *vanA* mobilome

European Journal of Clinical Microbiology & Infectious Diseases

Ewa Wardal, Dorota Żabicka, Waleria Hryniewicz and Ewa Sadowy

Dr Ewa Sadowy

Department of Molecular Microbiology, National Medicines Institute, Warsaw, Poland

E-mail address: [e.sadowy@nil.gov.pl](mailto:e.sadowy@nil.gov.pl)

Supplementary Table 2. Plasmids harbouring Tn*1546* among *E. faecalis* isolates submitted to GenBank.

| Accession number | Replicon type ^a,b^ | Plasmid size (kb) | Description | Tn*1546* structure ^c^ | Host ^b^ | | Country of isolation ^b^ | Year ^b^ | Reference |  |
| --- | --- | --- | --- | --- | --- | --- | --- | --- | --- | --- |
|  | Inc*18* |  |  |  |  | |  |  |  |  |
| CP060803.1 | *rep1* | 46.5 | *E. faecalis* SF28073 plasmid pSF3, complete sequence | wt | human (urine) | | US | 2003 | 1 |  |
| GQ484954.1 | *rep1* | 42.6 | *E. faecalis* plasmid pWZ909,  complete sequence | wt | human (wound) | | US | NA | 2 |  |
| GQ484955.1 | *rep1* | 47.3 | *E. faecalis* plasmid pWZ7140,  complete sequence | wt | human (colonization) | | US | NA | 2 |  |
| GQ484956.1 | *rep1* | 48.4 | *E. faecalis* plasmid pWZ1668,  complete sequence | wt | human (wound) | | US | NA | 2 |  |
| LR962527.1 | *rep2* | 72.9 | *E. faecalis* isolate 28157_4#381  genome assembly, plasmid: 2 | IS*256* between *vanX* and *vanY* | human (NA) | | Portugal | 2001 | 3 |  |
| LR962419.1 | *rep2* | 72.8 | *E. faecalis* isolate 28157_4#218  genome assembly, plasmid: 2 | IS*256* between *vanX* and *vanY* | human (urine) | | Portugal | 2007 | 3 |  |
| LR962506.1 | *rep2* | 32.0 | *E. faecalis* isolate 28099_2#54  genome assembly, plasmid: 5 | IS*1216* upstream *vanR* (deletion of transposase and resolvase genes) | human (exudate) | | Portugal | 2010 | 3 |  |
| LR961926.1 | *repUS1* | 107.6 | *E. faecalis* isolate 26975_2#147  genome assembly, plasmid: 2 | transposase gene truncated at 3’-end | bovine | | The Netherlands | 1996 | 3 |  |
| LR962379.1 | *repUS1* | 76.0 | *E. faecalis* isolate 28157_4#70  genome assembly, plasmid: 2 | IS*1216* upstream *vanR* (deletion of transposase and resolvase genes) | human (colonization) | | Brazil | 2016 | 3 |  |
|  | RepA_N |  |  |  |  | |  |  |  |  |
| LR962357.1 | *rep9b* | 61.3 | *E. faecalis* isolate 28157_4#90  genome assembly, plasmid: 2 | wt | human (bone) | | Brazil | 2015 | 3 |  |
| LR962389.1 | *rep9b* | 52.9 | *E. faecalis* isolate 28157_4#65  genome assembly, plasmid: 4 | wt | human (urine) | | Brazil | 2016 | 3 |  |
| LR962368.1 | *rep9b* | 62.9 | *E. faecalis* isolate 28157_4#86  genome assembly, plasmid: 2 | IS*1675* between transposase and resolvase genes | human (NA) | | Brazil | 2015 | 3 |  |
| LR962823.1 | *rep9b* | 63.5 | *E. faecalis* isolate 28157_4#78  genome assembly, plasmid: 3 | IS*1216* and IS*3* upstream *vanR* (deletion of transposase and resolvase genes) | human (colonization) | | Brazil | 2015 | 3 |  |
| LR962773.1 | *rep9c* | 88.9 | *E. faecalis* isolate 27688_1#300  genome assembly, plasmid: 2 | wt | human (NA) | | Hungary | NA | 3 |  |
| CP036247.1 | *rep9c* | 75.1 | *E. faecalis* R712 plasmid pR712_01, complete sequence | IS*L3* between *vanS* and *vanH* | human (blood) | | NA | NA | 4 |  |
|  | Multireplicons |  |  |  |  | |  |  |  |  |
| AB563188.1 | *rep1, rep9c* | 85.1 | *E. faecalis* plasmid pTW9,  complete sequence | wt | NA | | Taiwan | NA | unpublished |  |
| LR962268.1 | *rep2, rep7a* | 88.2 | *E. faecalis* isolate 27725_1#12  genome assembly, plasmid: 2 | wt | human (blood) | | Spain | 1999 | 3 |  |
| CP022486.1 | *repUS1, rep9c* | 31.4 | *E. faecalis* ARO1/DG plasmid pARO1.3, complete sequence | wt | dog (mastitis) | | New Zealand | 2001 | unpublished |  |
| LR962278.1 | *rep2, rep7a, rep9b* | 88.9 | *E. faecalis* isolate 27725_1#319  genome assembly, plasmid: 2 | wt | human (blood) | | Italy | 1993 | 3 |  |
| LR961971.1 | *rep7a,* *rep9b* | 95.6 | *E. faecalis* isolate 26975_2#148  genome assembly, plasmid: 2 | wt | human (respiratory tract) | | Belgium | 1997 | 3 |  |
| LR961954.1 | *rep7a, repUS43, rep9b* | 106.4 | *E. faecalis* isolate 26975_1#18  genome assembly, plasmid: 2 | wt | poultry | | The Netherlands | 1997 | 3 |  |
| **CP075608.1** | ***repUS1, rep18a*** | **21.6** | ***E. faecalis* strain 1207/14 plasmid p1207_4, complete sequence** | **IS*1216* upstream *vanR* (deletion of transposase and resolvase genes)** | **human (colonization)** | | **Poland** | **2014** | **5; this study** |  |
|  |  |  |  |  |  |  |  |  |  |  |
| AB247327.1 | NA | 128.1 | *E. faecalis* plasmid pSL1,  partial sequence | IS*1216* between *vanX* and *vanY* | human (NA) | Korea | | NA | 6 | |

The complete Tn*1546* was used as a query for blastn search of the GenBank nt/nr database (as of 29^th^ October 2021), limited by organism: “Enterococcus faecalis (taxid:1351)”, yielding 22 hits with > 50% query coverage. ^a^ replicon type determined using PlasmidFinder at <https://cge.cbs.dtu.dk/services/PlasmidFinder/> (29^th^ October 2021, date last accessed); ^b^ NA, data not available; ^c^ wt, wild-type Tn*1546* structure. Data on the 1207/14 isolate in bold.

References

1. Chatterjee A, Johnson CN, Luong P, Hullahalli K, McBride SW, Schubert AM, Palmer KL, Carlson PE Jr, Duerkop BA (2019) Bacteriophage resistance alters antibiotic-mediated intestinal expansion of enterococci. Infect Immun 87(6):e00085-19. doi: 10.1128/IAI.00085-19
2. Zhu W, Murray PR, Huskins WC, Jernigan JA, McDonald LC, Clark NC, Anderson KF, McDougal LK, Hageman JC, Olsen-Rasmussen M, Frace M, Alangaden GJ, Chenoweth C, Zervos MJ, Robinson-Dunn B, Schreckenberger PC, Reller LB, Rudrik JT, Patel JB (2010) Dissemination of an *Enterococcus* Inc18-like *vanA* plasmid associated with vancomycin-resistant *Staphylococcus aureus*. Antimicrob Agents Chemother 54(10):4314-4320. doi: 10.1128/AAC.00185-10
3. Pöntinen AK, Top J, Arredondo-Alonso S, Tonkin-Hill G, Freitas AR, Novais C, Gladstone RA, Pesonen M, Meneses R, Pesonen H, Lees JA, Jamrozy D, Bentley SD, Lanza VF, Torres C, Peixe L, Coque TM, Parkhill J, Schürch AC, Willems RJL, Corander J (2021) Apparent nosocomial adaptation of *Enterococcus faecalis* predates the modern hospital era. Nat Commun 12(1):1523. doi: 10.1038/s41467-021-21749-5.
4. Khan A, Davlieva M, Panesso D, Rincon S, Miller WR, Diaz L, Reyes J, Cruz MR, Pemberton O, Nguyen AH, Siegel SD, Planet PJ, Narechania A, Latorre M, Rios R, Singh KV, Ton-That H, Garsin DA, Tran TT, Shamoo Y, Arias CA (2019) Antimicrobial sensing coupled with cell membrane remodeling mediates antibiotic resistance and virulence in *Enterococcus faecalis*. Proc Natl Acad Sci USA 116(52):26925-26932. doi: 10.1073/pnas.1916037116.
5. Wardal E, Sadowy E (2021) Complete genome sequence of a Polish *Enterococcus faecalis* *vanA*-positive hospital isolate. Microbiol Resour Announc 10:e00668-21. https://doi.org/10.1128/MRA.00668-21
6. Lim SK, Tanimoto K, Tomita H, Ike Y (2006) Pheromone-responsive conjugative vancomycin resistance plasmids in *Enterococcus faecalis* isolates from humans and chicken feces. Appl Environ Microbiol 72(10):6544-6553. doi: 10.1128/AEM.00749-06.
